# Commentary: Target Intestinal Microbiota to Alleviate Disease Progression in Amyotrophic Lateral Sclerosis

**DOI:** 10.29245/2572.942x/2017/6.1136

**Published:** 2017-06-12

**Authors:** Jun Sun

**Affiliations:** Department of Medicine, University of Illinois at Chicago 840 S Wood Street, Room 704 CSB, MC716, USA

Amyotrophic lateral sclerosis (ALS), also called Lou Gehrig’s disease, is a neuromuscular disease characterized by a progressive death of motor neurons and muscle atrophy. Most ALS patients die within 5 years of disease onset. Currently, treatment with the US Food and Drug Administration (FDA) approved drug, Riluzole, merely extends the patient’s life span for a few months. For the second time in history, FDA has approved a new drug edaravone specifically for the treatment of ALS in May 8, 2017. There is still a need to develop new treatments for alleviating the disease progression and improving the lives of ALS patients.

The gut is considered the second brain of the human being. It contains roughly the same amount of neurons as the spinal cord. The integrative function of gut-secreted hormones plays an important role in human physiology and pathophysiology. Emerging evidence has shown that altered intestinal homeostasis and microbiome contribute to a variety of neurological diseases, including autism and Parkinson’s disease^1–3^. However, very little is known about the roles of intestine and microbiome in ALS. Our lab is the first to discover the link between intestinal homeostasis and the disease progression in an ALS mouse model G93A^4,5^.

Our recent study, entitled “*Target Intestinal Microbiota to Alleviate Disease Progression in Amyotrophic Lateral Sclerosis*”, has revealed a promising link of dysbiosis and aberrant intestine to disease progression in an ALS mouse model with overexpression of human mutation gene superoxide dismutase 1 (*SOD1^G93A^*)^4^. Mouse models expressing ALS-linked SOD1 mutations (e.g. SOD1^G93A^) effectively recapitulate many features of the human disease, and have been extensively used to investigate pathogenic mechanisms of ALS^6^. The SOD1^G93A^ mice show dysbiosis with reduced butyrate-producing bacteria^5^. These changes occur in young G93A mice before ALS onset. In the current study, we hypothesize that restoring microbiome and intestinal homeostasis delays disease onset and progression of ALS. After being fed with the beneficial bacteria product butyrate, G93A mice exhibited a delay in the onset of ALS symptoms and a prolonged life span.

Bacterial products short chain fatty acids (SCFA) provide energy for the colonocytes and exert beneficial effects in the gut^7^. Butyrate (a SCFA) and butyrate-producing bacteria are thought to have beneficial effects to the host through anti-inflammatory properties^8^. In our study, treatment with butyrate delays disease onset in the SOD1^G93A^ mouse. Butyrate synthesis by anaerobic bacteria can occur via butyryl-coenzyme A (CoA): acetate CoA-transferase^9^. In our study, oral 2% sodium butyrate treatment for 2.5 months restores the physiological CoA enzyme levels (comparable to wild type mice) in pre-symptomatic SOD1 mice. Interestingly, our unpublished data also show that ALS patients had significantly low butyrate and total SCFA levels.

Intestinal epithelia of SOD1^G93A^ mice have a disrupted tight junction structure accompanied with increased intestinal permeability^4,5^. Paneth cells are specialized intestinal epithelial cells that regulate the host-bacterial interactions in gut^10,11^. The abnormal Paneth cells were significantly increased in the SOD1^G93A^ mice. We found that butyrate, restored some of the intestinal defects and corrected dysbiosis in the ALS mice. At the cellular level, butyrate treatment decreased abnormal Paneth cells. Moreover, butyrate reduced aggregation of the G93A-SOD1 mutated protein in small intestine and colon of ALS mice.

Based on the current data, the improvements due to pre-symptomatic butyrate treatment include improving the intestinal barriers, correcting dysbiosis, restoring Paneth cells, and reducing aggregation of the G93A-SOD1 mutated protein in ALS mice, thus slowing down the progression of disease. This study focuses on investigating the novel role of gut microbiome and intestinal functions in ALS and exploring potential therapeutic targets for ALS by restoring healthy host-bacterial interactions. It highlights the complex roles of microbial and intestinal homeostasis that contribute to the neuromuscular dysfunction in ALS. It has provided insight into targeting intestinal functions and microbiome that may slow down ALS pathogenesis.

Sodium phenylbutyrate has been widely considered as an inhibitor of histone deacetylases (HDAC)^12,13^. It is used in the ALS mice through intraperitoneal injection^14^ and tested in ALS patients for safety^15^. Sodium phenylbutyrate can contribute to gut function through modulation of tight junctions^16^. Therefore, the proactive role of sodium phenylbutyrate in ALS could also through restoring intestinal permeability. Understanding the molecular mechanism for the direct beneficial roles of butyrate on neuromuscular dysfunction in ALS is an ongoing study in our research team. A HDAC inhibitor could be used as a control for its effects on intestinal microbiome.

Most ALS cases are sporadic (SALS), with about 10% being familial (FALS). Both SALS and FALS manifest similar pathological and clinical phenotypes, suggesting that different initiating causes lead to a mechanistically similar neurodegenerative pathway. A fraction of FALS are associated with mutations in the superoxide dismutase gene *SOD1*^17^. It may be interesting to evaluate other ALS experimental models, i.e. transactive response DNA binding protein (TDP)-43 mouse model).

In SALS, there is study reported the increased circulating bacterial lipopolysaccharides (LPS) and systemic immune activation^18^. Our unpublished data evaluates the gastrointestinal health and microbiome profile of patients with heterogenous motor neuron syndromes. We observed decreased diversity, altered ratio of two major bacterial members: *Firmicutes* and *Bacteroidetes*, and low butyrate in human ALS. Only one patient did not have a low *Firmicutes* / *Bacteroidetes* ratio. Further, our unpublished study has shown elevated intestinal inflammation in ALS patients. Along these lines, ALS G93A mice showed abnormal intestinal microbiome, in which butyrate-producing bacteria (*Butyrivibrio* Fibrisolvens) were reduced. *Firmicutes* make up the largest portion of the mouse and human gut microbiome^19^. The division *Firmicutes* as part of the gut flora has been shown to be involved in energy resorption and obesity^20,21^. *Fimicutes Peptostreptococus*, which was low in ALS mice^5^, was also enhanced after butyrate treatment. Several clinical studies have demonstrated the safety for butyrate or its derivative in human subjects^22^. Thus, our study with oral sodium butyrate treatment in the G93A mice may be applied to both SALS and FALS for translation to clinical therapy.

Humans have coevolved with their microbes over thousands of years, but this relationship, is now being dramatically affected by shifts in the collective human microbiome resulting from changes in the environment and societal norms^23^. Mismatches in host-microbe relationships lead to homeostatic chaos, likely explaining the increased incidence and prevalence of many disorders that have merged with alarming frequency in the modern age. We still lack research on the early intestinal issue from ALS patients. ALS research need learn from the experience and lessons in the filed of inflammatory bowel diseases, obesity, and other chronic diseases. A better understanding of human microbiome would facilitate the development of targeted interventions to control the progression of ALS.
